# Highly Conductive and Flexible Gel Polymer Electrolyte with Bis(Fluorosulfonyl)imide Lithium Salt via UV Curing for Li-Ion Batteries

**DOI:** 10.3390/membranes9110139

**Published:** 2019-10-30

**Authors:** Lei Jin, Faiz Ahmed, Taewook Ryu, Sujin Yoon, Wei Zhang, Yonghoon Lee, Daeho Kim, Hohyoun Jang, Whangi Kim

**Affiliations:** 1 Department of Applied Chemistry, Konkuk University, 268 Chungwon-daero, Chungju-si 27478, Chungcheongbuk-do, Korea; jinlei8761@naver.com (L.J.); faiz2310@gmail.com (F.A.); gundam0924@naver.com (T.R.); ysj920126@naver.com (S.Y.); arno_zw@hotmail.com (W.Z.); jedo50@naver.com (Y.L.); wskdh2004@naver.com (D.K.); 2 Department of Liberal arts, Konkuk University, 268 Chungwon-daero, Chungju-si 27478, Chungcheongbuk-do, Korea; 200417450@kku.ac.kr

**Keywords:** gel polymer electrolytes, UV curing, lithium bis(fluorosulfonyl)imide, room temperature ionic conductivity

## Abstract

A series of new self-standing gel polymer electrolytes (SGPEs) were fabricated by ultraviolet (UV) curing and investigated for application in flexible lithium-ion batteries. Compared with traditional gel polymer electrolytes (combine with solvents or plasticizers), these new SGPEs were prepared simply by curing different weight ratios of lithium bis(fluorosulfonyl)imide (LiFSI) with a methacrylic linear monomer, poly (ethylene glycol) dimethacrylate (PEGDMA). Noticeably, there were no solvents or plasticizers combined with the final SGPEs. Owing to this, the SGPEs showed high flexibility and strong mechanical stability. Some paramount physicochemical and electrochemical characters were observed. The SGPEs demonstrated good thermal stability below 150 °C and an extremely low glass transition temperature (*T_g_*) (around −75 °C). Moreover, plastic crystal behaviors were also identified in this study. Ultimately, the SGPEs demonstrated excellent ionic conductivity at room temperature, which proves that these new SGPEs could be widely applied as a prospective electrolyte in flexible lithium-ion batteries.

## 1. Introduction

Lithium-ion batteries (LIBs) have been paid increasing attention in recent decades (since 1991) due to their high energy density, long cycle lives, and high efficiency. Meanwhile, they are widely applied in various mobile devices, electric vehicles, and energy storage areas [1,2,3,4]. Similarly to other types of battery, there are three indispensable components in an LIB: anode, cathode, and electrolyte. The electrolyte plays a critical role in transporting lithium ions between the pair of electrodes and determines the speed of energy release [5,6]. Organic liquid electrolytes have been commercialized for decades, and currently take up a large proportion of the batteries market owing to their excellent ambient temperature ionic conductivity. However, safety issues caused by leakage and flammability of organic liquid have become an obstacle to the development of LIBs; such safety issues cannot be ignored anymore and must be resolved as soon as possible [7,8,9,10]. Recently, solid polymer electrolytes (SPEs) have been introduced as alternative to traditional organic liquid electrolytes able to solve the aforementioned issues [11,12]. Extensive studies have shown that SPEs not only possess outstanding safety properties, but also suppress the growth of lithium dendrites. Moreover, SPEs can also be utilized as separators in LIBs because of their excellent mechanical stability [13,14,15]. Generally, SPEs are composed of polymer and lithium salts; lithium ions from the lithium salts can transfer through the free volume provided by the polymer [16]. To date, various materials have been reported and researched as polymer hosts, including poly (ethylene oxide) (PEO), poly (acrylonitrile) (PAN), poly (methyl methacrylate) (PMMA), and poly (vinylidene fluoride-hexafluoro propylene) (PVdF-HFP), etc. [17]. However, poor ambient temperature ionic conductivity (10^−5^ S/cm) of SPEs, which is caused by a high Ohmic drop in the electrolyte, is the main barrier limiting their wide application in LIBs [18,19,20,21]. To cope with this barrier, high-performance gelled polymer electrolytes (GPEs) have received great interest and have been investigated by some groups. Traditional GPEs are usually composed of SPEs and plasticizers or organic solvents which are utilized in liquid electrolytes; hence, GPEs can provide high ionic conductivity (10^−3^ S/cm) close to that of organic liquid electrolytes at room temperature and simultaneously maintain excellent mechanic stability similarly to SPEs [14,17,22]. However, it is also generally accepted that the mechanical stability of GPEs is sacrificed if the concentration of plasticizer or organic solvent is increased, even if the ionic conductivity is improved [23]. Meanwhile, GPEs combined with plasticizers and organic solvents in high concentrations can lead to bulky battery packages, and the leakage and flammability issues caused by organic solvents become a concern again [24]. Consequently, safe and high-performance electrolytes are still required.

To overcome the aforementioned issues, in this research, a series of high-ionic-conductivity self-standing gel polymer electrolytes (SGPEs) were fabricated by UV curing. UV curing is widely applied in the coatings industry for protecting the surface of various substrates. UV curing enables quick and simple polymerization and is quite environmentally friendly, besides which it allows formulation without any solvents. Furthermore, the glazed and smooth appearance which results from UV curing also could offer favorable interfacial contact with electrodes [25,26]. For the polymer matrix, a low-molecular-weight UV-curable poly (ethylene glycol) dimethacrylate (PEGDMA) was introduced as a new polymer matrix. PEO and its derivatives (PEGDMA) usually possess exceedingly high dielectric constants, resulting in high solubility of the lithium salts. The ethylene oxide units in PEGDMA offer a high donor number for alkali metals, so the Li ions can easily form a coordination linkage with them. With this coordination linkage (Li–O) forming and breaking, Li ions can jump along the PEGDMA chains to achieve ion transport. In addition, PEGDMA also has high chain flexibility owning to the low rotational energy barrier between oxygen and methylene, which is beneficial for the transport of Li ions. As a result, the high chain flexibility of PEGDMA is expected to improve the ionic conductivity [27,28]. For the lithium salt, LiFSI has been extensively studied as an electrolyte salt because of its high ionic conductivity comparable to LiPF_6_, and superior stability towards hydrolysis over LiPF_6_. Furthermore, LiFSI (0.45 ppm Cl^−^) demonstrates better stability with an Al current collector than LiTFSI [29]. These merits are can be attributed to the medium-range anion size of the FSI^−^ group, which exhibits promising chemical stability and dissociation [29,30,31,32]. LiFSI can also offer a significant impact in terms of decreasing the crystallinity of the electrolyte [33,34]. Finally, anhydrous acetone was added to obtain homogenous precursor electrolyte solutions; however, this was removed completely before the UV-curing process.

In order to compare the effect of the LiFSI on the SGPE properties, various weight ratios of SGPE were prepared. All the SGPEs were investigated by Fourier-transform infrared spectroscopy (FT–IR), thermal gravimetric analysis (TGA), differential scanning calorimetry (DSC), and X-ray diffraction (XRD), while the ionic conductivities were determined by electrochemical impedance spectroscopy (EIS). Finally, the ion transference number was measured to confirm the cations’ mobility in the investigated electrolytes.

## 2. Experiment

### 2.1. Materials

**Figure 1:**
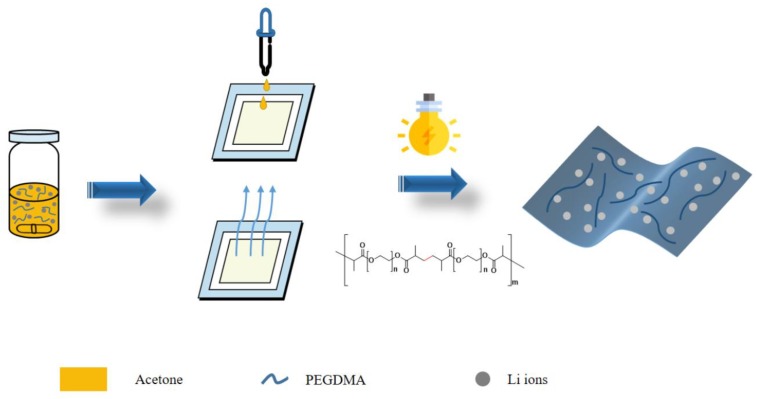
The process of fabricating a self-standing gel polymer electrolyte (SGPE) by UV curing. PEGDMA: poly (ethylene glycol) dimethacrylate.

The chemical reagents, including polyethylene glycol dimethacrylate (PEGDMA, average Mn 550), photoinitiator 2-Hydroxy-2-methylpropiophenone (HMPP, 97%), and lithium hexafluorophosphate solution in ethylene carbonate and dimethyl carbonate (50v/50v) (1.0 M LiPF_6_ in EC/DMC) were purchased from Aldrich (Seoul, South Korea). LiFSI was obtained from Chunbo Co., Ltd. (Chungju, South Korea). A Hg UV lamp (600 W) with an irradiation peak intensity of 200 mW/cm^2^ was purchased from Lichtzen (Gunpo, South Korea). The acetone solvent was purified by distillation to remove water. The UV polymerization process and chemical structure are described in Figure 1.

### 2.2. Fabrication of Self-Standing Gel Polymer Electrolytes

The SGPE precursor solutions were prepared by dissolving different weight ratios of LiFSI into PEGDMA. Photoinitiator HMPP was then added into the solutions, wherein the concentration of HMPP was 1–2 wt.% of the amount of PEGDMA. Anhydrous acetone was added finally to obtain a homogenous solution, wherein the ratio of acetone and mixture solution was 3:1 (v/w). Afterward, the mixture was stirred at room temperature for ~3–4 h until all the starting materials had dissolved. The precursor solution was cast onto a glass plate (8 cm × 8 cm) attached to a silicon mold (6 cm × 6 cm, inside), and anhydrous acetone was removed in a vacuum oven at 40 °C. Finally, the electrolyte modeling precursor was exposed to Hg UV lamp irradiation for 20 min to obtain a self-standing gel polymer electrolyte. All the SGPEs were fabricated through the same process. Herein, the “s” in SGPE sample names describes the wt.% of LiFSI in the electrolytes.

### 2.3. Characterization of Self-Standing Gel Polymer Electrolytes

**Figure 2:**
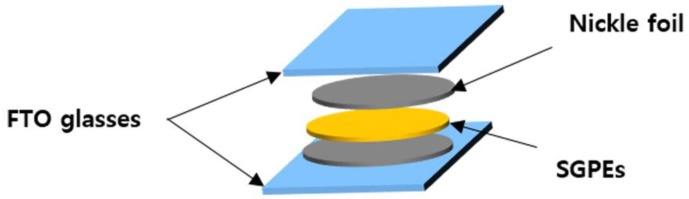
Illustration of the homemade cell assembly.

The polymerization of the SGPEs was confirmed by FT–IR (Nicolet iS5 FTIR Spectrometer, Waltham, MA, US) with a spectral resolution of 4 cm^−1^ at room temperature. Thermal stability behaviors were determined using a thermal gravimetric analyzer (Perkin-Elmer TGA-7, Massachusetts, US) from 25 °C to 500 °C at a heating rate of 10 °C/min under a nitrogen flow rate of 10 mL/min. The weight of the samples was controlled between 3 and 5 mg. Glass transition temperatures (*T_g_*) of the SGPEs were detected by DSC (Perkin-Elmer DSC 6000, Waltham, MA, US) from −80 to 120 °C with a heating rate of 10 °C/min under a nitrogen gas atmosphere. XRD (Dmax2500/PC, Tokyo, Japan) was utilized to verify the plastic crystal behavior of the SGPEs and PEGDMA. The samples were scanned between 10 and 80° with a speed rate of 2°/min at room temperature. In order to calculate the ionic conductivity (*σ*, mS/cm), EIS was done, primarily with an IM6ex, Zahner- Elektrik GmbH & Co. KG instrument (Kronach, Germany) from 30 to 90 °C and over a frequency range of 0.1 Hz to 1 MHz with an amplitude of 5 mV. All the EIS spectra were fitted with an appropriate equivalent circuit model using Z-view software (version 3.1, Scribner Associates Inc., US). To assemble a homemade cell (Figure 2), the SGPEs were sandwiched between two disks of conductive nickel foil and positioned between two FTO (Fluorine doped tin oxide) glass electrodes. The conductivity was calculated using Equation (1):

*σ* = *l*/(*R* × *A*) (1)

where *l* and *A* are the thickness and the area of the SGPEs, respectively. *R* is the resistance value of the SGPEs.

Ion transference number (*t*_Li+_) was measured with a symmetrical Swagelok cell Li/SGPEs/Li which was sealed inside an Ar-filled glove box, and was calculated in this work according to Equation (2):

*t*_Li+_ = *I_s_* (Δ*V* – *I*_0_*R*_0_)/*I*_0_ (Δ*V* – *I_s_R_s_*) (2)

where *I*_0_, *I_s_*, *R*_0_, *R_s_*, and Δ*V* are the initial current, steady-state current, applied polarization voltage, initial resistances of passivation layers, and the steady-state resistances of passivation layers, respectively.

## 3. Results and Discussion 

**Figure 3:**
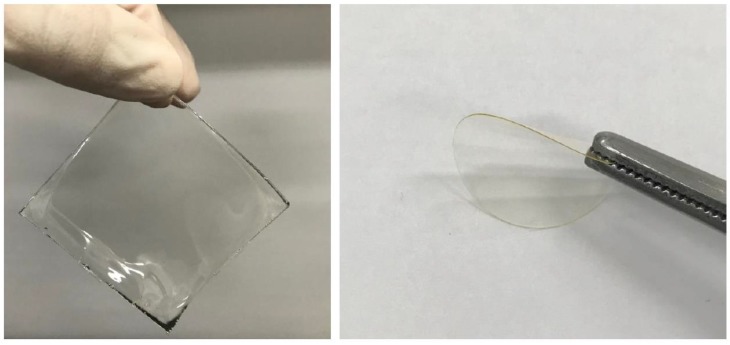
The physical appearance of the SGPEs at room temperature.

**Figure 4:**
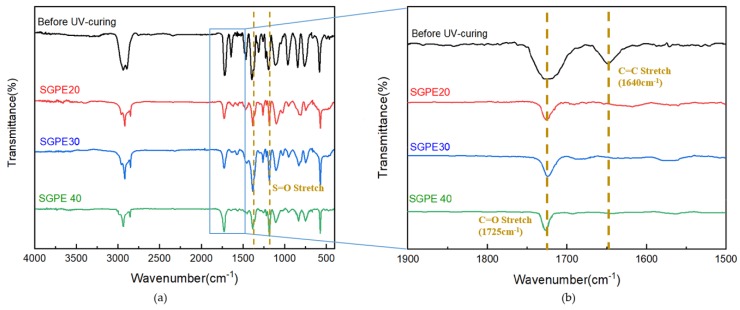
FT–IR spectra of (**a**) SGPEs before/ after UV curing and (**b**) acrylic C=C bonds in the SGPEs before/ after UV curing.

A series of SGPEs were fabricated with the precursor mixture solutions through UV-curing polymerizations, and the appearances of SGPEs are presented in Figure 3. The precursor solutions turned to transparent and flexible electrolyte films which could readily be peeled away from glass plates. The thickness of the SGPEs was controlled under 300 µm by adjusting the silicon mold size and the amount of precursor solutions. SGPE50 is not presented here as its excessive lithium salt content led to the inability to fabricate an intact electrolyte film. The chemical structure and UV polymerization of the SGPEs were confirmed by FT-IR. Before UV curing, the PEGDMA characteristic absorption peaks of the C=C and C=O double bonds were present at 1640 cm^−1^ and 1725 cm^−1^, respectively, as shown in Figure 4. After UV curing, only the C=O double bonds remained at 1725 cm^−1^, while the peak of the C=C double bond disappeared. Furthermore, S=O stretch peaks typical of LiFSI were located at 1376 cm^−1^ and 1172 cm^−1^ whether before UV curing or after. The FT–IR graph suggests that polymerization of SGPEs was achieved after UV curing and that LiFSI was still present after the curing process.

**Figure 5:**
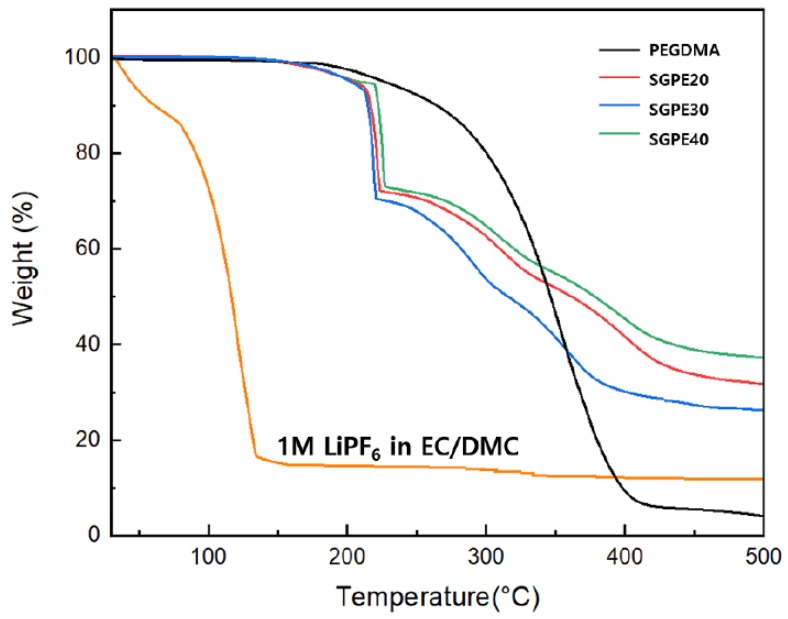
Thermal stability of pure PEGDMA, SGPEs, and 1M LiPF6 in ethylene carbonate and dimethyl carbonate (EC/DMC).

Heat is produced when batteries operate, and overheating is one of the most common causes of LIB accidents. As a result, all of the components in batteries require favorable thermal stabilities, including electrolytes and separators [35,36]. Thermal characteristics were detected by TGA and DSC, respectively. It is shown in Figure 5 that pure PEGDMA possessed the best thermal stability, with weight degradation beginning from 175 °C. In contrast, the commercial liquid electrolyte (1.0 M LiPF_6_ in EC/DMC) displayed the lowest heat endurance, and its weight declined sharply from the beginning. In the case of the SGPEs, weight loss began slightly at 150 °C and dramatically increased from 210 °C, which corresponded with the thermal stability of LiFSI and PEGDMA. Figure 5 indicates that compared with commercial liquid electrolyte, the SGPEs possess more promising thermal stability, which could improve the battery thermal stability and safety.

**Figure 6:**
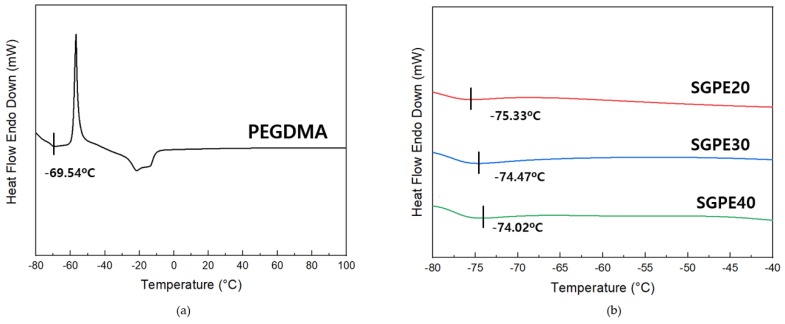
Glass transition temperatures of (**a**) PEGDMA and (**b**) SGPEs.

According to Vogel–Tammann–Fulcher behavior, the *T_g_* of a polymer is related to its free volume and ionic conductivity. Generally, ionic conductivity shows a reciprocal relationship with *T_g_*; decreasing the *T_g_* of the material increases the ionic conductivity of the electrolyte [37,38]. In Figure 6, the SGPEs presented extremely low *T_g_*: −75.33 °C (SGPE20), −74.47 °C (SGPE30), and −74.02 °C (SGPE40)—significantly lower than pure PEGDMA (−69.54 °C). A small increase was seen with the decline of PEGDMA concentration, but *T_g_* remained at around −74 °C. In addition, there was no endothermic peak observed with increased temperature, indicating that the SGPEs have no melting point. The DSC graph demonstrates that after UV curing, amorphous SGPEs with excellent *T_g_* were obtained. Due to the different concentrations of LiFSI in the SGPEs, slight fluctuations of *T_g_* were observed, but the plastic crystal behaviors of the SGPEs were not disrupted.

**Figure 7:**
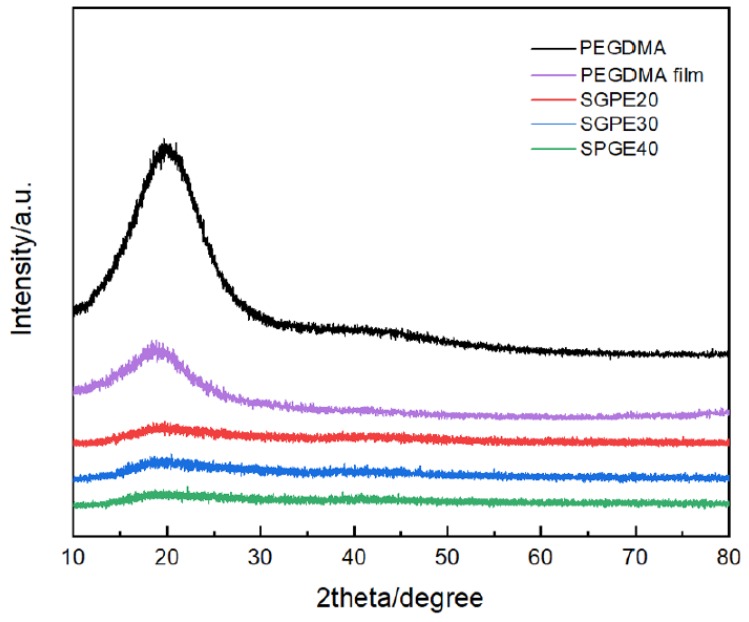
X-ray diffraction (XRD) pattern of pure PEGDMA, PEGDMA film, and SGPEs.

As mentioned above, the crystal phase is considered as the main factor that retards polymer chain dynamics and leads to low ion transportation. This phenomenon can be relieved by the use of an amorphous phase with activated chain segments [27]. The plastic crystal behaviors of PEGDMA and SGPEs were confirmed by XRD analysis. As shown in Figure 7, it was apparent that pure PEGDMA monomer displayed a big broad peak at 20°, which corresponded to the crystalline property of PEGDMA. In contrast to this, after UV curing, this typical peak was dramatically declined to a tiny one for the SGPEs, confirming that amorphous SGPEs had been obtained. Moreover, the SGPEs also demonstrated a reduced crystal phase compared to the pure PEGMDA film, suggesting that LiFSI suppressed the crystal phase considerably.

**Figure 8:**
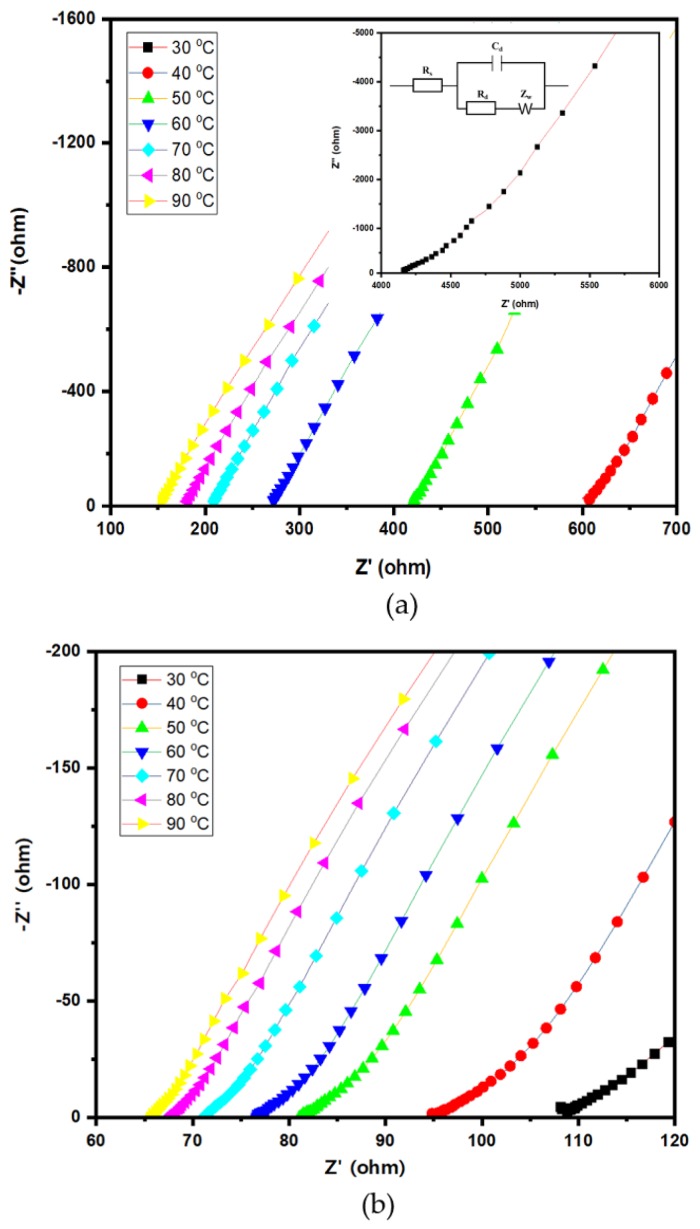
Nyquist plots of (**a**) SGPE30 and (**b**) SGPE40.

**Figure 9:**
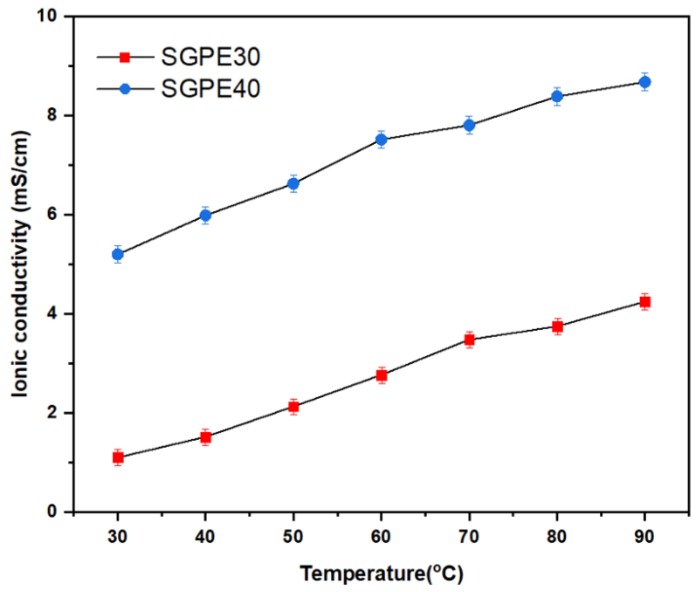
Temperature-dependent ionic conductivity of SGPE30 and SGPE40.

**Figure 10:**
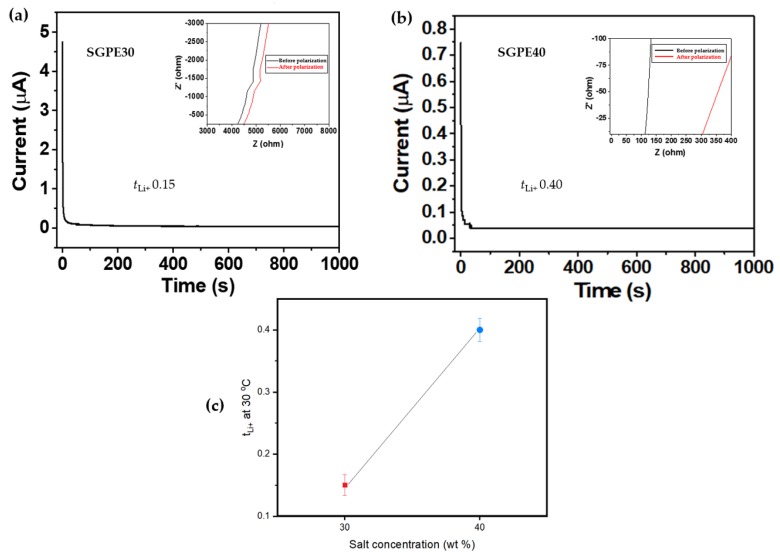
*t*_Li+_ values for (**a**) SGPE30, (**b**) SGPE40, and (**c**) dependence of *t*_Li+_ on salt concentration for SGPEs at 30 °C.

The ionic conductivity of electrolytes is one of the most critical factors for LIBs, indicating the movement ability of lithium ions in an electrolyte. It is also the main barrier to commercialization of polymer electrolytes in LIBs. In order to study the ionic conductivity of the SGPEs, EIS is carried out at different temperatures from 30 to 90 °C, and the Nyquist plots are displayed in Figure 8. Note that only SGPE30 and SGPE40 are discussed in this section, since the impedance of SGPE20 was too high to be used as a reference. It is obvious that SGPE40 had lower resistance compared to SGPE30; this trend applied to the whole measured temperature range. According to Equation (1), these results can directly affect the ionic conductivities of SGPEs. As shown in Figure 9 and Table 1, both SGPE30 (*σ* = 1.11 mS/cm) and SGPE40 (*σ* = 5.21 mS/cm) reached a favorable level of ionic conductivity at room temperature, extremely close to that of the the liquid electrolyte. Additionally, SGPE40 was observed to have higher ionic conductivity than that of SGPE30 throughout the investigated temperature range. In other words, at a given temperature, SGPE40 had superior Li-ionic conduction to SGPE30. This may imply that in a network system, ionic conductivity can be improved by increasing the amount of LiFSI. All the SGPE ionic conductivities climbed moderately with the growth of temperature. The lithium-ion transference number of unit indicated that the ion transport and rate performance of the battery were dominated exclusively by lithium ions [24]. Many attempts have been made to improve the transference number of polymer electrolytes to meet the requirements of LIBs. According to the report, crystalline PEO-based electrolytes usually offer quite low transference numbers (<0.20), even at Li salt concentrations greater than 30 mol.% [39]. Figure 10a, b shows the chronoamperometry curves of the symmetrical Swagelok Li/SGPEs/Li cells at a polarization voltage of 10 mV, and the inset of Figure 10 shows the Nyquist plots of the dummy cell before and after polarization. Figure 10c displays the values of *t*_Li+_ dependent on LiFSI concentration for SGPEs; the values of *t*_Li+_ increased with increasing concentration of LiFSI up to 0.40 at a concentration of 40 wt.%, which is higher than general PEO-based electrolytes. However, SGPE30 only reached 0.15 due to its low concentration of LiFSI.

## 4. Conclusion

In this work, different weight ratios of LiFSI was used as the lithium salt in fabrication of a highly conductive and flexible polymer electrolyte for LIBs. The electrolyte polymerization was carried out by UV curing at room temperature, and it was observed that a high lithium salt concentration (≥50%) made it difficult to form an intact electrolyte film by this method. Thus, SGPE20, SGPE30, and SGPE40 were investigated in detail. The SGPEs displayed satisfactory thermal stability below 150 °C and extremely low *T_g_* (around −75 °C), demonstrating that the concentration of LiFSI had a very slight effect on the thermal properties of SGPEs. Additionally, all the SGPEs displayed similar plastic crystal behaviors (amorphous) after the UV-curing reaction. Excellent ionic conductivity of both SGPE30 (1.11 mS/cm) and SGPE40 (5.21 mS/cm) were observed at room temperature; the impedance of SGPE20 was so high that it could not be measured here. Moreover, different lithium-ion transference numbers were confirmed for SGPE30 (0.15) and SGPE40 (0.40), implying that ionic conductivity and transference numbers were effectively increased by enhancing the concentration of LiFSI. Summarized above, this work demonstrated the breakthrough that a self-standing gel polymer electrolyte based on PEGDMA and LiFSI can be a candidate electrolyte polymer for LIB applications. Comprehensive battery tests will need to be conducted for this type of electrolyte in order to move towards commercialization.

## Figures and Tables

**Table 1:** Ionic conductivity of solvent-free electrolyte at different temperatures.

Sample	30 °C	40 °C	50 °C	60 °C	70 °C	80 °C	90 °C
SGPE30	1.11^1^	1.52	2.13	2.77	3.48	3.75	4.25
SGPE40	5.21	5.99	6.63	7.52	7.81	8.39	8.38

^1^ mS/cm.
